# Projection of Stroke Incidence and Costs in China: 2022 to 2050

**DOI:** 10.1161/STROKEAHA.125.054522

**Published:** 2026-08-18

**Authors:** Yutong Jiang, Lizheng Xu, Xia Wang, Meiqiyang Xue, Mingsheng Chen, Lei Si

**Affiliations:** School of Health Policy and Management, Nanjing Medical University, China (Y.J., M.X., M.C.).; Healthcare Big Data Research Institute, School of Public Health, Cheeloo College of Medicine, Shandong University, Jinan, P.R. China (L.X.).; The George Institute for Global Health, Faculty of Medicine, University of New South Wales, Sydney, Australia (X.W.).; School of Health Sciences, Faculty of Health, Western Sydney University, Campbelltown, New South Wales, Australia (L.S.).; Translational Health Research Institute, Western Sydney University, Penrith, New South Wales, Australia (L.S.).

**Keywords:** China, cost of illness, forecasting, healthcare costs, incidence, ischemic stroke, stroke

## Abstract

**BACKGROUND:**

Stroke imposes a substantial and growing health and economic burden in China, yet forward-looking estimates of stroke-related costs remain scarce.

**METHODS:**

Annual age- and sex-specific incidence rates of ischemic stroke, intracerebral hemorrhage, and subarachnoid hemorrhage were projected from 2022 to 2050 using 2021 as the baseline year. Baseline incidence estimates were obtained from the Global Burden of Disease 2021 database, and future trends were projected using an exponential growth model parameterized by the average annual percent change estimated from 1990 to 2021 Global Burden of Disease data. Projected incidence rates were combined with China-specific population projections. A deterministic decision-tree model was used to estimate hospitalization costs, direct medical costs, and societal costs over a 12-month time horizon in China, using probabilities and unit costs from published literature. We used uncertainty intervals (UI) to quantify the plausible range of all projected outcomes. One-way sensitivity analyses were conducted to assess uncertainty in epidemiological and cost parameters.

**RESULTS:**

Total incident strokes are projected to increase from 4.35 million (95% UI, 3.09–5.89 million) in 2022 to 9.59 million (95% UI, 6.81–13.01 million) in 2050. By 2050, ischemic stroke, intracerebral hemorrhage, and subarachnoid hemorrhage incidences are projected to reach 7.89, 1.59, and 0.11 million, respectively. Total annual stroke hospitalization costs are projected to rise from $46.1 billion (95% UI, 44.94–47.24 billion) in 2022 to $91.2 billion (95% UI, 89.28–93.13 billion) in 2050; direct medical costs and societal costs are projected to increase from $67.6 billion to $133.9 billion and from $145.6 billion to $288.4 billion, respectively. Costs will be increasingly concentrated among adults aged ≥65 years, especially those aged ≥85 years. In 2050, stroke costs among men are projected to be approximately 1.3 times those for women. In sensitivity analyses incorporating incidence uncertainty, total annual hospitalization costs ranged from $39.9–$62.3 billion in 2022 to $65.0–$123.6 billion in 2050.

**CONCLUSIONS:**

Stroke incidence and costs in China are projected to increase through 2050, driven primarily by ischemic stroke and population aging, highlighting the need for targeted prevention, capacity planning for older adults, and long-term financing strategies.

Stroke remains a leading cause of morbidity, disability, and mortality worldwide. In recent decades, the burden of stroke has shifted from developed to developing countries, where 75% of stroke deaths and 81% of stroke-related disability-adjusted life years occur.^1^ Globally, the absolute number of incident strokes increased by 70% from 1990 to 2019, reaching 12.2 million new cases in 2019, comprising 7.63 million ischemic strokes (ISs), 3.42 million intracerebral hemorrhages, and 1.18 million subarachnoid hemorrhages.^2^ Projections suggest that the annual global incidence will continue to rise, reaching 14.69 million new stroke cases per year by 2030 and 21.43 million per year by 2050.^3^ Beyond its health impact, stroke also imposes a major economic burden, with global costs estimated at approximately 890 billion (United States dollars [USD]; ≈0.66% of global gross domestic product).^4^

China accounts for a large share of the global stroke burden. According to China’s stroke surveillance report, there were an estimated 17.8 million prevalent stroke survivors and 3.4 million new stroke cases annually, with stroke-related expenditures exceeding 58 billion Chinese yuan in 2020.^5^ According to the Global Burden of Diseases (GBD) Study 2021, China has the highest number of ischemic stroke cases, and deaths among countries, accounting for over 33% of global stroke-related disability-adjusted life years.^6^ With rapid population aging and evolving stroke epidemiology, forward-looking estimates of inpatient hospitalization costs are needed to inform prevention strategies, hospital capacity planning, and sustainable financing. Although several studies have projected future stroke incidence or disability burden,^6–8^ there is limited evidence on how annual stroke-related costs will evolve by stroke type, age, and sex in China.

Using an exponential growth model based on GBD 2021 incidence trends and a deterministic decision-tree cost model, this study projected age- and sex-specific incidence and annual costs for IS, intracerebral hemorrhage, and subarachnoid hemorrhage in China from 2022 to 2050. Hospitalization costs were estimated primarily from the hospital perspective, and broader direct medical and societal costs were estimated over a 12-month time horizon. The objectives were to quantify future fiscal pressures, support evidence-based resource allocation, and inform long-term policies that prioritize cost-effective prevention in an aging society.

## Methods

This study was reported in accordance with the CHEERS (Consolidated Health Economic Evaluation Reporting Standards) 2022 statement^9^ (completed checklist in Supplemental Material).

### Data Availability Statement

All data analyzed in this study are publicly available from open-access sources. Access to the data could be made available upon reasonable request to the corresponding author.

### Ethics Statement

This study analyzed public, aggregated, and deidentified data and did not involve human participants, human tissue, or individually identifiable information; therefore, ethics approval and informed consent were not applicable.

### Projection of Annual Stroke Incidence

We focused on 3 stroke types: IS, intracerebral hemorrhage, and subarachnoid hemorrhage, as they represent the majority of stroke cases and differ in pathophysiology,^8^ clinical outcomes, and healthcare costs. Stroke incidence rates categorized by type, sex (male, female, and total), and 5-year age groups (0–4, 5–9,..., 80–84, and ≥85 years) in the GBD 2021 database from 1990 to 2021 were used to calculate the average annual percent change (AAPC) for each category. The year 2021 was the baseline year for this analysis. Future incidence trends (2022–2050) were projected annually for sex, age, year, and stroke type using an exponential growth model:


$$\begin{array}{c} IR_{t}=IR_{2021}\times {\left(1+AAPC\right)}^{t-2021}, \end{array}$$

where *IR*_*t*_ denotes the incidence rate in year *t*; $IR_{2021}$ denotes the incidence rate in the baseline year (2021); AAPC denotes the AAPC in incidence rate estimated from 1990 to 2021 GBD data; and t−2021 denotes the length of the projection period. Projected annual case numbers for each stroke type were calculated by multiplying projected incidence rates by the corresponding age-, sex-, and year-specific population projections. Population projections were sourced from the China’s Population Projection-Medium Variant report, jointly prepared by the China Population and Development Research Center and the United Nations Population Fund China.^10^ Age- and sex-specific population projections and AAPC estimates are presented in Tables S1 and S2, respectively.

### Cost Estimation

We developed a deterministic expected-value decision-tree model to capture costs for the index hospitalization and, conditional on survival to discharge, a subsequent readmission within 1 year. Three mutually exclusive outcomes were considered: discharge without readmission, discharge with readmission, and in-hospital death (Figure). For patients discharged alive, total costs included the index hospitalization expenditure plus, if readmitted, the recurrent stroke cost; for in-hospital death, we applied an in-hospital death cost. Readmission was defined as stroke readmission after a stroke hospitalization.^11^ For each stroke type and calendar year, projected incident cases entered the same decision tree independently. The decision tree itself did not include repeated annual cycles. Time was incorporated by applying the annual projected number of incident cases from 2022 to 2050 and by estimating costs over a 12-month postindex event time horizon. This model was used for the base-case cost estimation and 1-way sensitivity analyses.

**Figure.**
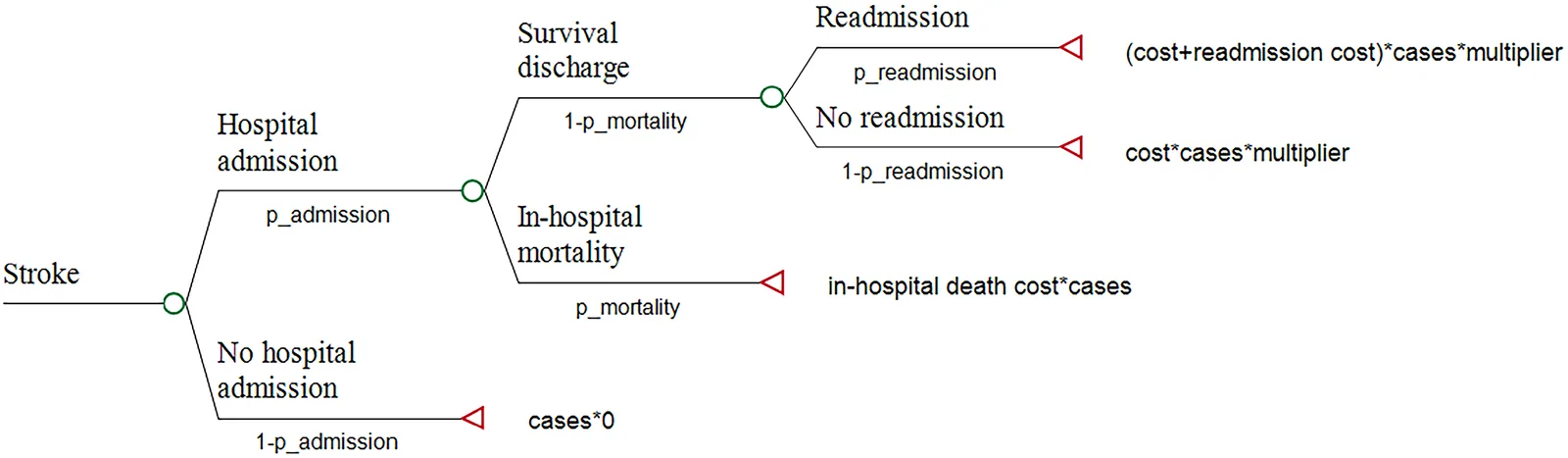
Decision-tree model for incident costs estimation of stroke patients.

To identify model input parameters, we conducted targeted literature searches in PubMed and Web of Science from database inception to January 2026. The literature search and screening process is presented in Figure S1. Search terms included combinations of “stroke,” “ischemic stroke,” “intracerebral hemorrhage,” “subarachnoid hemorrhage,” “China,” “cost,” “hospitalization cost,” “inpatient cost,” “readmission,” “recurrence,” “mortality,” and “cost of illness.” We prioritized studies conducted in China that reported stroke-type-specific hospitalization costs, in-hospital mortality, readmission or recurrence rates, and 1-year cost components compatible with our decision-tree structure. Studies were excluded if they did not provide extractable parameter estimates, were not specific to China, did not distinguish relevant stroke types, did not clearly describe the composition of costs, or focused on selected treatment subgroups only. Although many studies described stroke burden or costs, only a small number provided parameters compatible with the model structure and perspective. The overall methodological framework and key assumptions are summarized in Table S3. Transition probabilities and unit costs were obtained from the published literature and summarized in Table 1. Inpatient costs represent total in-hospital expenditures per admission as reported in the source studies and include prescribed medicines, rehabilitation therapy, surgeries, prescription drugs, imaging examinations, medical consumables, beds, laboratory investigations, diagnostic and nursing expenses.^12^ We estimated total costs from the hospital perspective, healthcare system perspective, and societal perspective over a 12-month time horizon. Direct medical costs and societal costs were derived by applying literature-derived multipliers to hospitalization costs.^15^ These multipliers capture additional postdischarge costs (rehabilitation and nursing), direct nonmedical costs, and indirect costs. The cost-component ratios used to derive direct medical and societal costs are presented in Table S4. Cost inputs were converted to 2021 USDs using the 2-stage computation adapted to the consumer price index and purchasing power parity.^19^ All projected costs are presented in 2021 USD.

**Table 1.**
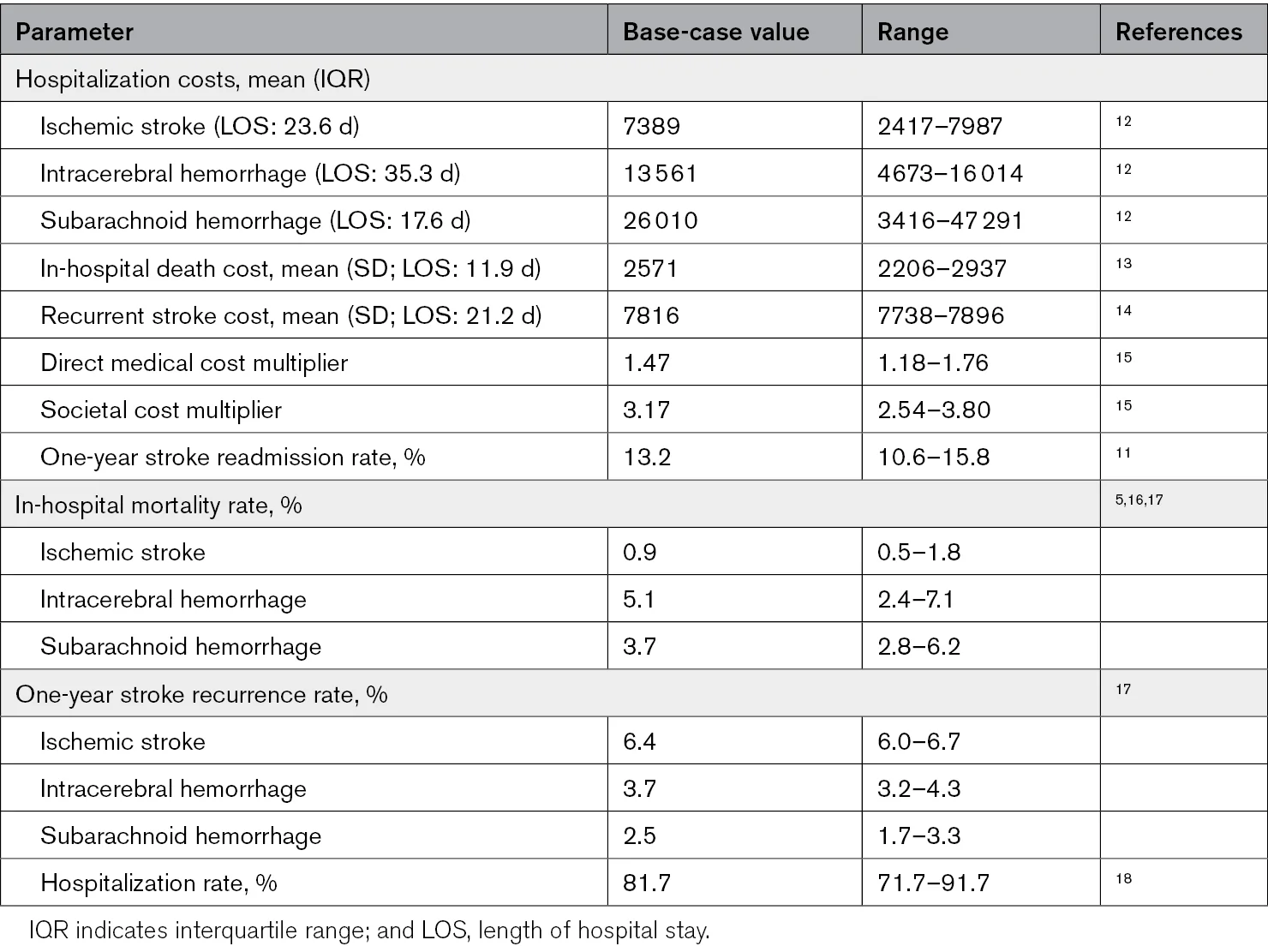
Key Parameters in the Decision-Tree Model

### Sensitivity Analysis

We conducted 1-way sensitivity analyses to assess how uncertainty in key parameters affects projected hospitalization costs. We varied the hospitalization rate, in-hospital mortality rates, and recurrence-related assumptions within the bounds shown in Table 1 and recalculated annual hospitalization costs for 2022 to 2050. For the hospitalization rate (derived from a national cross-sectional survey^18^), because the source study did not report uncertainty intervals, we applied a±10 percentage-point scenario bound to represent plausible variation in treatment-seeking and access. In an alternative scenario, we replaced stroke readmission rates with stroke-type-specific recurrence rates and applied the recurrent stroke cost to represent a more conservative estimate of postdischarge utilization. For broader economic perspectives, we varied the direct medical and societal cost multipliers within the reported ranges to quantify uncertainty in these cost estimates. GBD 2021 provides uncertainty intervals for incidence estimates. To reflect epidemiological uncertainty, we incorporated uncertainty in both the 2021 baseline incidence rate and the AAPC used for incidence projections. To evaluate potential distortion of the 2021 baseline incidence due to the COVID-19 pandemic, we repeated the incidence projections using 2019 (prepandemic) GBD incidence rates as the baseline year. Incidence-uncertainty scenarios and projections using the 2019 pre-COVID-19 baseline incidence rates are presented in Tables S5 and S6, respectively.

### Statistical Analysis

AAPCs were estimated using the Joinpoint Regression Program (Statistical Research and Applications Branch, National Cancer Institute). Data cleaning and incidence forecasting were performed in R 4.3.3 (R Foundation for Statistical Computing). Projected incidence case numbers were calculated using population projections in Microsoft Excel (Microsoft Office Professional 2021). TreeAge Pro 2022 was used for the base-case cost estimation and 1-way sensitivity analyses.

## Results

### Main Results

From 2022 to 2050 in China, the projected annual number of stroke cases increases substantially for IS, rising from 2965 thousand to 7892 thousand, with increases observed in both men (1654–4420 thousand) and women (1308–3455 thousand). In contrast, intracerebral hemorrhage shows a modest increase in incidence (1239–1586 thousand), whereas subarachnoid hemorrhage declines (147–111 thousand).

Consistent with these incidence patterns, costs are dominated by IS and are projected to grow markedly over time. Annual stroke hospitalization costs are projected to rise from $46.1 billion in 2022 to $91.2 billion in 2050. Using the same case projections and applying cost multipliers, total annual direct medical costs are projected to increase from $67.6 billion in 2022 to $133.9 billion in 2050, and total annual societal costs from $145.6 billion to $288.4 billion. IS hospitalization costs increase from $24.6 to 65.6 billion, direct medical costs from $36.2 to 96.4 billion, and societal costs from $78.0 to 207.7 billion by 2050. For intracerebral hemorrhage, costs rise moderately (hospitalization: $18.0–23.0 billion; direct medical: $26.3–33.7 billion; societal: $56.6–72.4 billion). In contrast, subarachnoid hemorrhage costs decrease across all components (hospitalization: $3.5–2.6 billion; direct medical: $5.1–3.8 billion; societal: $11.0–8.3 billion). In 2050, stroke costs among men are projected to be ≈1.28 × those among women across all cost perspectives. Projected annual stroke cases and costs are presented in Table 2. Detailed age- and sex-specific incident case projections and annual hospitalization costs are presented in Tables S7 and S8, respectively. Age-specific trends for IS, intracerebral hemorrhage, and subarachnoid hemorrhage and changes in the age-group composition of incident stroke cases are shown in Figures S2 through S5.

**Table 2.**
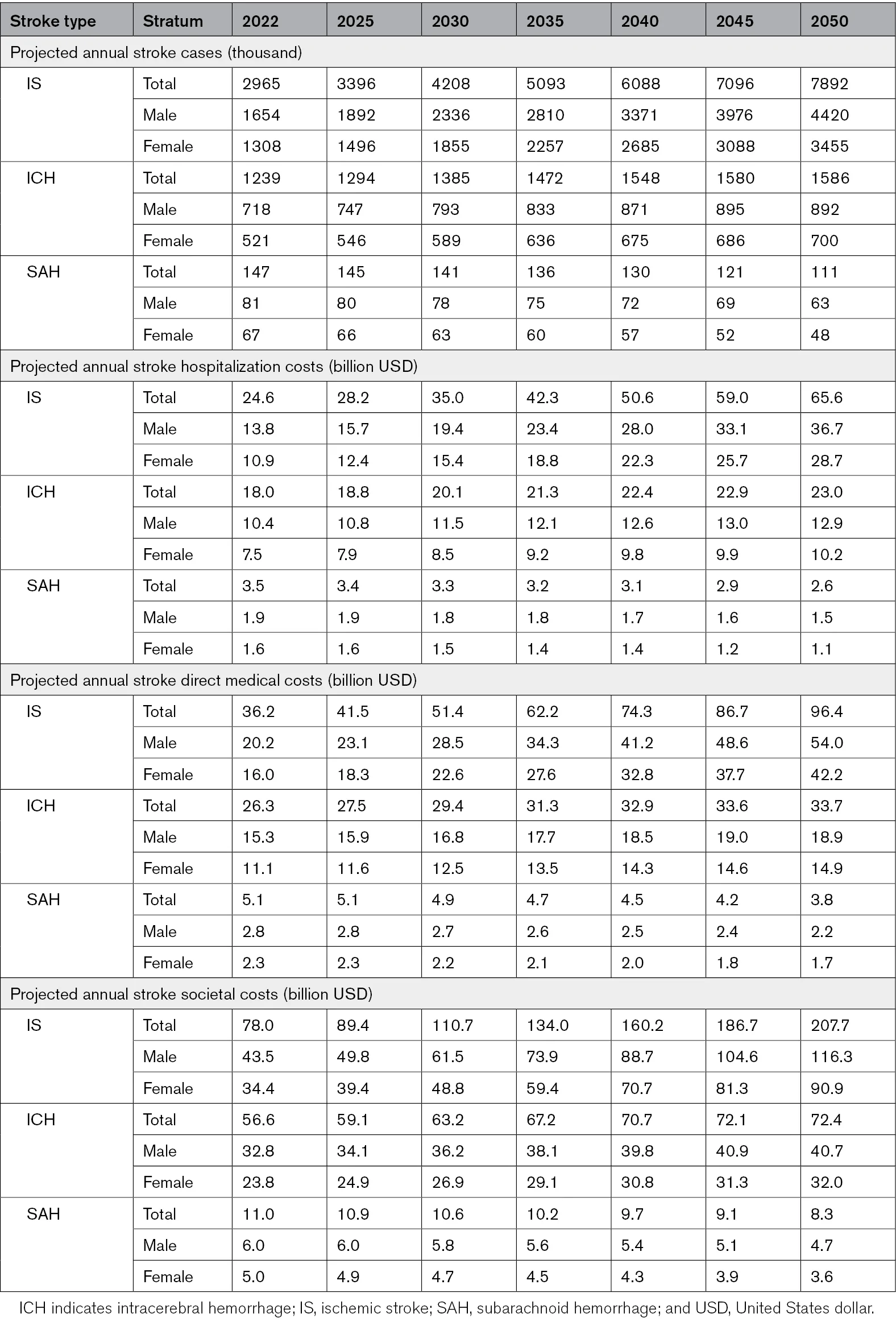
Projected Annual Stroke Cases and Costs by Sex in China (2022–2050)

Across age groups, projected stroke hospitalization costs are highly age-dependent and increasingly concentrated among older adults. Costs are minimal in children and adolescents (generally <$0.1 billion per age band) but rise steeply after age 50 years, with the largest absolute burden consistently observed in those aged ≥70 years, particularly those aged ≥75 years. Specifically, costs for those aged <55 years are projected to decline, while costs for the 55–64 age group rise modestly. The greatest absolute growth occurs in the oldest age groups: hospitalization costs for those aged ≥85 years increase from $6.17 billion in 2022 to $21.68 billion in 2050. Because direct medical and societal costs were derived by scaling hospitalization costs, their age and sex distributions follow the same pattern. These age-specific results are presented in Table 3.

**Table 3.**
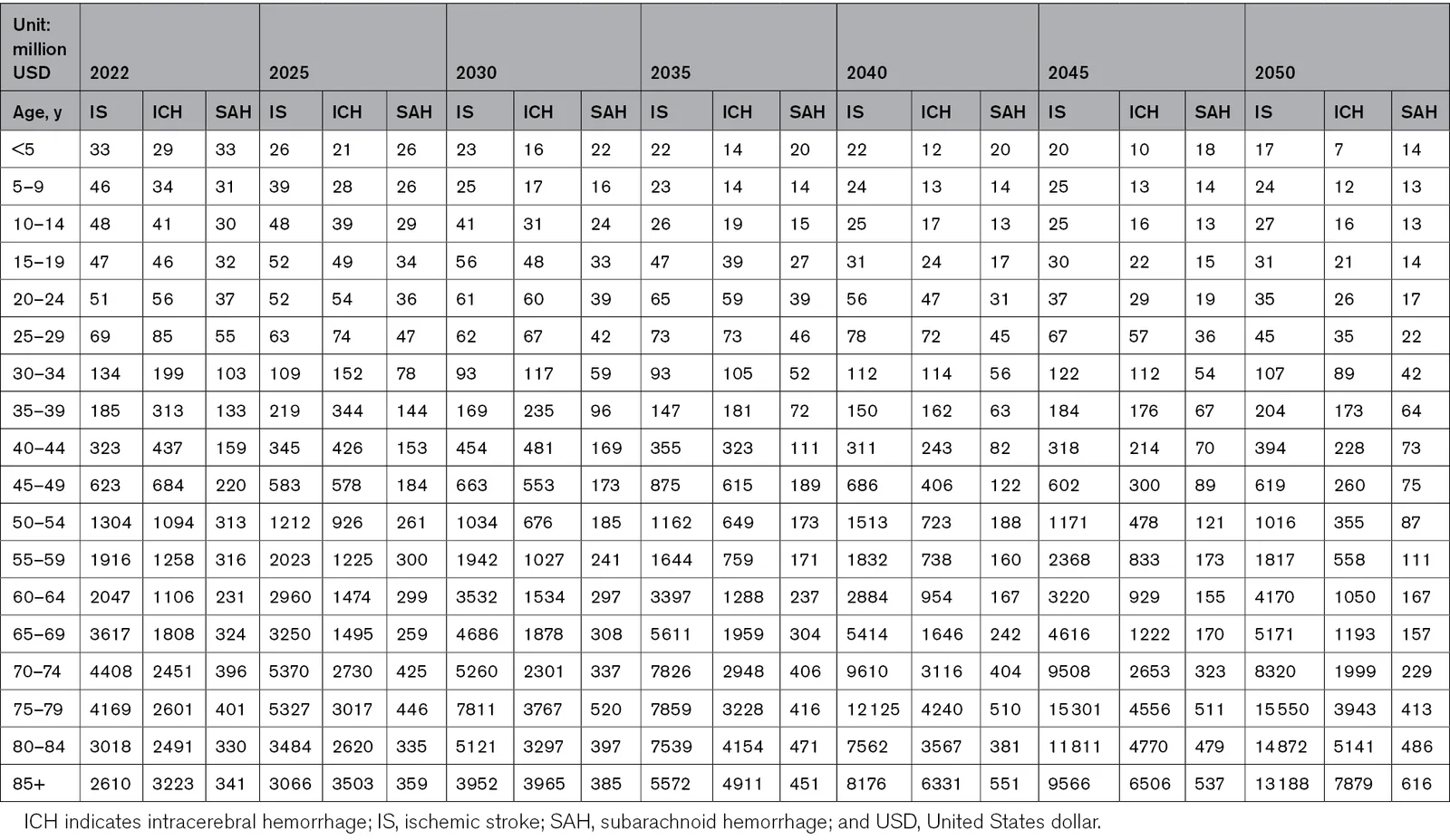
Projected Annual Stroke Hospitalization Costs by Age Group in China (2022–2050)

### Sensitivity Analysis

Table 4 presents 1-way sensitivity analyses for projected annual stroke costs. Varying the hospitalization rate yields total annual hospitalization costs ranging from $33.4–$42.7 billion in 2022 to $66.2–$84.5 billion in 2050. Varying in-hospital mortality rates within reported bounds produces a comparatively narrow range ($46.2–$47.2 billion in 2022; $91.5–$92.9 billion in 2050). Replacing the stroke readmission rate with stroke-type-specific recurrence rates yields lower projected costs ($43.9–$44.3 billion in 2022; $86.4–$87.1 billion in 2050). For broader economic perspectives, uncertainty in the direct medical cost multiplier results in total annual direct medical costs of $55.1–$82.0 billion in 2022 and $108.7–$162.0 billion in 2050. Uncertainty in the societal cost multiplier yields total annual societal costs of $118.1–$176.7 billion in 2022 and $233.6–$349.4 billion in 2050. In an additional scenario applying uncertainty to projected incidence, total annual hospitalization costs ranged from $39.9–$62.3 billion in 2022 to $65.0–$123.6 billion in 2050. Overall, hospitalization rate and postdischarge utilization assumptions introduce greater variability than in-hospital mortality. Detailed incidence-uncertainty scenarios are presented in Table S5. Projections using the 2019 pre-COVID-19 baseline incidence rates are presented in Table S6.

**Table 4.**
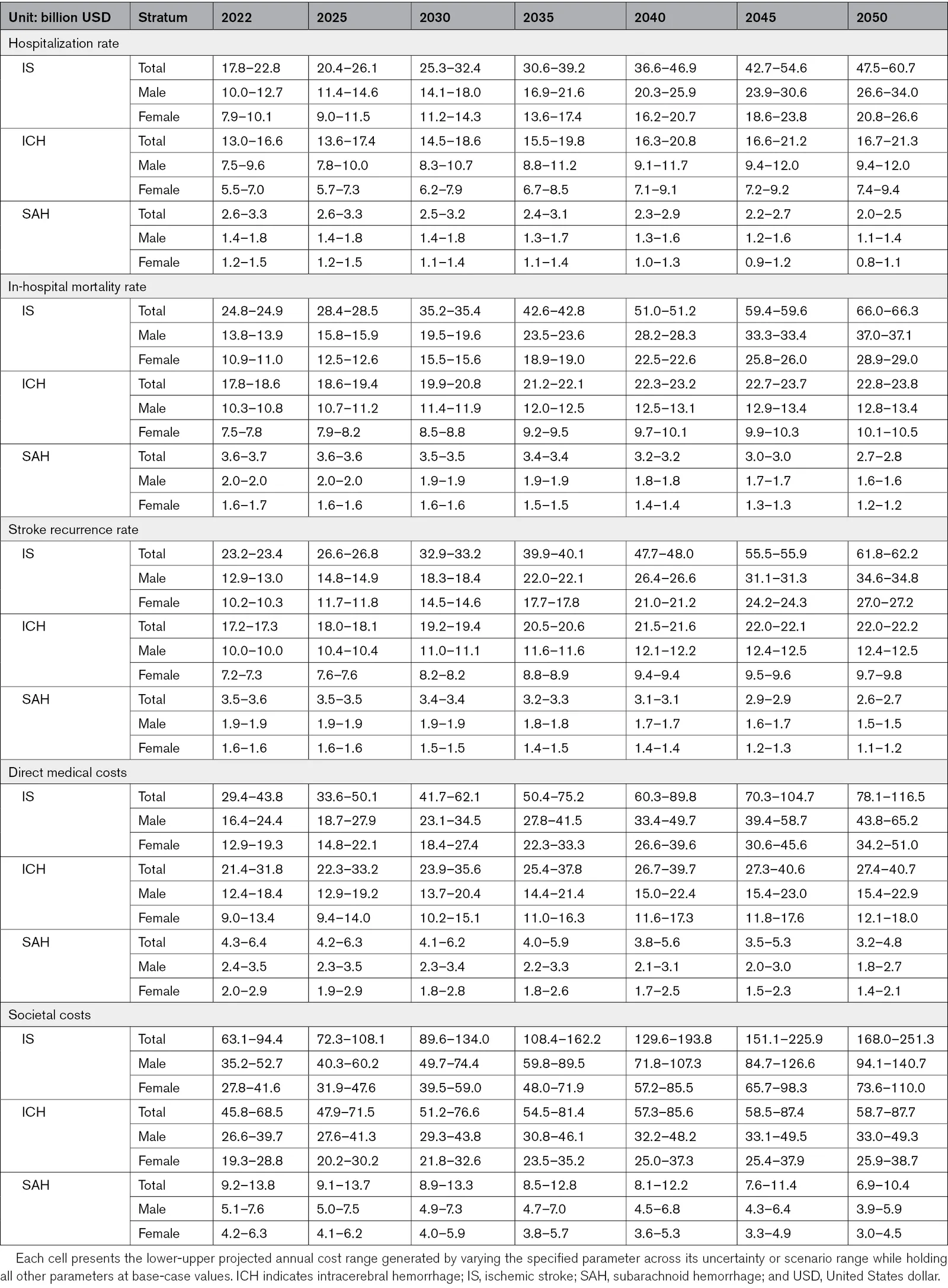
One-Way Sensitivity Analyses for Projected Annual Stroke Costs in China (2022–2050)

## Discussion

This study projected stroke incidence using GBD 2021 incidence trends, while the predicted costs were estimated using the stroke incidence multiplied by the nationally endorsed population in China in the respective years. The total number of incident strokes was projected to increase from 4.35 million in 2022 to 9.59 million in 2050 (about +120%). Correspondingly, annual hospitalization costs were projected to nearly double from $46.1 billion in 2022 to $91.2 billion in 2050. When costs beyond the index hospitalization were considered over a 12-month horizon, total annual direct medical costs were projected to rise from $67.6 billion to $133.9 billion and total annual societal costs from $145.6 billion to $288.4 billion. The burden will be increasingly concentrated in older adults. People aged ≥65 years accounted for 69.8% of hospitalization costs in 2022 and were projected to account for 86.8% by 2050, with the steepest growth in those aged ≥85 years ($6.17–$21.68 billion). Costs among men are projected to remain higher than those among women (≈1.28-fold in 2050).

Rising stroke-related costs represent a common trend in aging populations worldwide. In the United Kingdom, annual stroke events were projected to rise by 60% between 2015 and 2035, and stroke prevalence to increase by 120%.^20^ Societal costs were expected to nearly triple, with healthcare and social-care costs rising by 201% and 273%, respectively.^20^ In the United States, adult stroke prevalence is projected to increase from 3.9% in 2020 to 6.4% in 2050, alongside rises in hypertension (51.2%–61.0%) and diabetes (16.3%–26.8%), and stroke is expected to contribute the largest absolute increase in cardiovascular-related healthcare costs.^21,22^ By contrast, a Swedish longitudinal study found that declines in first and recurrent stroke incidence were large enough to offset population aging, indicating that stronger prevention can moderate future burden.^23^ Overall, these studies indicate that aging, improved survival, and persistent vascular risk factors increasingly shift stroke burden toward older adults in need of long-term rehabilitation and support. This is especially important in the wider neurological disease landscape: neurological conditions were the leading global cause of ill health and disability in 2021, with stroke and dementia among the major contributors,^24^ while dementia-attributable healthcare spending is projected to rise from USD 263 billion in 2019 to USD 1.6 trillion by 2050.^25^ Stroke is therefore likely to compete increasingly with dementia and other neurodegenerative disorders for rehabilitation capacity, community services, long-term care, and caregiver time, underscoring the need for integrated planning across acute, rehabilitative, and long-term care systems.

The projected increase in stroke cases will be driven primarily by IS. The incidence of IS is projected to rise by 166% between 2022 and 2050, and it accounts for ≈72% of total costs across all perspectives by 2050. Hospitalization costs for IS increase from $24.6 billion in 2022 to $65.6 billion in 2050, and 1-year societal costs from $78.0 billion to $207.7 billion. These patterns likely reflect rapid population aging and the growing prevalence of vascular risk factors such as hypertension, diabetes, and atrial fibrillation.^26–29^ A previous projection study on IS also found that the incidence rate will increase in both sexes and across all age groups.^30^ The estimated proportion of IS in 2050 (82.3%) is similar to that in 2020 (86.8%),^31^ with similar proportions observed in recent years.^5^ Given current trends, IS costs are projected to rise rapidly over the next 30 years.^20^

Although IS is projected to drive most of the future growth in stroke burden, intracerebral hemorrhage should not be neglected. Our projections show that intracerebral hemorrhage remains a substantial contributor to hospitalization costs in China through 2050, despite a smaller number of cases than IS. This indicates that the future stroke burden will not be limited to IS alone, and intracerebral hemorrhage will continue to demand considerable healthcare resources. From a policy perspective, these findings highlight the importance of maintaining adequate capacity for intracerebral hemorrhage prevention, acute care, and hospital resource planning, rather than concentrating resources solely on IS. The projected decline in subarachnoid hemorrhage cases results from a long-term downward trend in age- and sex-specific subarachnoid hemorrhage incidence rates observed in the GBD historical series. This trend may reflect improvements in modifiable risk factors, such as better hypertension control and reduced smoking prevalence, which have contributed to declining subarachnoid hemorrhage incidence in recent decades.^32^ When extrapolated to future projections, this negative incidence trend is strong enough to offset demographic growth from population aging, yielding fewer projected subarachnoid hemorrhage cases in absolute terms.

Male hospitalization costs are projected to remain higher than female costs primarily because men have higher age-specific stroke incidence rates in the underlying GBD series, leading to more projected admissions. We did not assume sex-specific differences in per-admission costs or length of stay; therefore, sex differences in spending in this analysis are largely epidemiologically driven. This higher male incidence aligns with established epidemiological patterns, associated with a greater prevalence of key modifiable risk factors including smoking, hypertension, heavy alcohol use, and adverse lipid profiles, as reported in population-based stroke studies in China and worldwide.^33,34^ In the real world, additional factors including sex differences in stroke severity, comorbidity burden, care-seeking behavior, and access to postacute rehabilitation could also influence resource use and spending. Future work using large administrative data sets could examine these sex-specific utilization patterns in greater detail.

The divergent age pattern in projected hospitalization costs likely reflects demographic change and age-specific stroke risk. In our model, total costs are mainly driven by projected incident cases and average expenditures per hospital admission. Thus, the decline in costs among individuals aged <55 years is primarily attributable to fewer projected stroke cases, driven by shrinking younger population cohorts. In contrast, costs rise significantly in older age groups because stroke risk is concentrated at older ages, particularly among those aged ≥65 years. Moreover, the projected shift of costs toward older groups is consistent with prior work showing that demographic change is a dominant driver of China’s future stroke burden.^23,35,36^ These findings suggest that the healthcare system in China is expected to prepare for increasing demand for acute stroke care, rehabilitation, and long-term support, especially for older adults.

By extending beyond inpatient costs, our estimates also show that the broader economic burden of stroke is substantially larger than hospitalization costs alone. Using multipliers from a national 1-year cost study, hospitalization costs represent only part of the total direct medical and societal burden. This finding is consistent with prior cost-of-illness studies reporting that inpatient care accounts for only a minority of total stroke-related expenditures.^31^ Therefore, planning based solely on inpatient budgets may underestimate the full economic consequences of stroke, particularly the growing need for postdischarge services and caregiver support.

Our projections are also consistent with historical GBD evidence from China showing that even when age-standardized stroke incidence has declined or stabilized, the absolute number of strokes can continue to rise because of rapid population aging.^37^ Type-specific patterns are similarly aligned with long-run trends, with IS increasing most, intracerebral hemorrhage remaining a substantial contributor, and subarachnoid hemorrhage declining.^38^ Together, these findings suggest that demographic momentum will continue to drive substantial growth in the absolute burden and costs of stroke in China over the coming decades.

A major strength of this study is the use of standardized age- and sex-specific incidence estimates from GBD 2021 combined with nationally endorsed population projections, allowing the forecasts to explicitly reflect demographic change in China. In addition, hospitalization costs were estimated using a decision-tree model that incorporated major in-hospital pathways and literature-based probabilities and unit costs. We also conducted sensitivity analyses to quantify the impact of uncertainty in hospitalization, recurrence, and mortality assumptions.

This study has several limitations. First, we assumed that hospitalization costs remained constant over time. However, medical price inflation, technological changes, and evolving standards of care could influence future costs.^39^ This may lead to underestimation of the projected total costs. Second, our inpatient unit costs were sourced from published literature. Although China-specific stroke cost studies are increasing, studies remain geographically limited and may not fully capture geographic heterogeneity across China, urban-rural differences, insurance schemes, hospital levels, and patient characteristics. Only studies that provided extractable and model-compatible parameters were used for the final decision-tree inputs. Third, our model employs average per-admission costs by stroke type, which may not fully capture age-related variations in severity, comorbidity, and requirements for rehabilitation or nursing care. Fourth, direct medical and societal costs were derived using multipliers from a 1-year cost study of IS and were applied to hemorrhagic stroke types;^15^ in addition, in the absence of specific cost data for recurrent strokes, we used average annual hospitalization costs as a proxy for recurrent-event costs. These assumptions may introduce bias. Fifth, we relied on GBD 2021 incidence estimates, which synthesize heterogeneous data sources using statistical modeling. Although GBD provides standardized estimates and uncertainty intervals, the estimates may be affected by differences in surveillance systems, case ascertainment, diagnostic coding, data availability, and modeling assumptions across locations and time periods.^40,41^ These limitations may influence both the baseline incidence and AAPC used in our projections and should be considered when interpreting projected stroke cases and costs. Sixth, several uncertainty ranges were based on the available published data rather than patient-level distributions. For parameters without reported uncertainty intervals, particularly the hospitalization rate, we used a ±10 percentage-point scenario range to explore plausible variation in treatment-seeking and access. This should be interpreted as a scenario assumption rather than a statistically estimated CI. Finally, the decision-tree model captured 1 readmission within 12 months and in-hospital death but did not include multiple recurrent events, longer-term outpatient care, or disability beyond 1 year; therefore, lifetime costs were not estimated.

Our estimates represent a baseline projection under continuation of historical incidence trends and current average inpatient expenditure patterns. Future scenario analyses could quantify the potential cost offsets from proven interventions, such as improved population blood pressure control, detection and anticoagulation for atrial fibrillation, strengthened tobacco control, and wider access to evidence-based acute reperfusion therapy and stroke unit care.

## Conclusions

Our projections indicate a substantial increase in stroke incidence and costs in China from 2022 to 2050, driven primarily by IS and population aging. Annual hospitalization, direct medical, and societal costs are projected to nearly double, with an increasing concentration of the burden among adults aged ≥65 years. These findings support strengthening primary prevention, improving early detection and management of vascular risk factors in older adults, and planning sustainable financing and service capacity for acute and postacute stroke care.

## ARTICLE INFORMATION

### Disclosures

None.

### Supplemental Material

Tables S1–S8

Figures S1–S5

CHEERS Checklist

## Supplementary Material


