# Supplementary material for: Projection of Stroke Incidence and Costs in China: 2022 to 2050
Source: Stroke. 2026 Aug 18;57(10):3081–90. doi: 10.1161/STROKEAHA.125.054522 (PMC13619140; doi:10.1161/STROKEAHA.125.054522)
Supplement: Supplementary file 1 [file str-57-3081-s001.pdf]

Supplemental Material

Supplement to: Projection of stroke incidence and costs in China: 2022-2050.

Contents:

Table S1. Age- and sex-specific population projections used in the model, China, 2022–2050 3

Table S2. Average annual percentage change in stroke incidence rates by sex and age group, 1990–2021 4

Table S3. Summary of methodological framework and key assumptions........ 6

Table S4. Empirical cost composition and cost ratios relative to hospitalization cost 8

Table S5. Sensitivity analysis of projected incident stroke cases in China, 2022–2050 (Unit: million) 9

Table S6. Projected incident stroke cases in China using 2019 (pre-COVID-19) baseline incidence rates, 2022-2050........ 10

Table S7. Projected age-specific incident stroke cases by stroke type and sex in China, 2022-2050........ 11

Table S8. Projected annual hospitalization costs of stroke by type and sex in China, 2022–2050 (Unit: billion) 14

Figure S1. Literature search and screening process for model input parameters 15

Figure S2. Projected incidence of ischemic stroke by age group, 2022–2050 16

Figure S3. Projected incidence of intracerebral hemorrhage by age group, 2022–2050 17

Figure S4. Projected incidence of subarachnoid hemorrhage by age group, 2022–2050 18

Figure S5. Projected trends in the proportions of stroke incidence by age group, 2022–2050 19

Consolidated Health Economic Evaluation Reporting Standards (CHEERS) Checklist 20

Table S1. Age- and sex-specific population projections used in the model, China, 2022–2050

Age / Year	0-4		5-9		10-14		15-19		20-24		25-29		30-34		35-39		40-44	
	**Male**	**Female**	**Male**	**Female**	**Male**	**Female**	**Male**	**Female**	**Male**	**Female**	**Male**	**Female**	**Male**	**Female**	**Male**	**Female**	**Male**	**Female**
**2022**	3246	2932	4700	4187	4789	4175	4172	3603	3920	3361	4432	3930	5946	5499	5543	5234	4946	4688
**2025**	2647	2411	4037	3638	4796	4223	4570	3976	3939	3377	4049	3514	4814	4328	6378	5998	5109	4825
**2030**	2469	2292	2641	2405	4033	3635	4791	4220	4562	3972	3930	3372	4038	3507	4797	4317	6343	5976
**2035**	2527	2364	2464	2287	2638	2403	4029	3633	4783	4216	4553	3967	3921	3367	4025	3499	4773	4303
**2040**	2690	2514	2522	2358	2462	2285	2636	2402	4023	3630	4775	4211	4543	3961	3909	3360	4007	3489
**2045**	2670	2494	2685	2509	2521	2357	2460	2284	2633	2400	4017	3626	4765	4205	4531	3953	3894	3350
**2050**	2401	2243	2666	2490	2683	2508	2519	2356	2457	2282	2629	2398	4010	3621	4755	4197	4516	3943
	**45-49**		**50-54**		**55-59**		**60-64**		**65-69**		**70-74**		**75-79**		**80-84**		**85+**	
**2022**	5212	5004	6264	6130	5772	5735	3517	3552	3806	3940	2729	2943	1621	1839	959	1189	677	1065
**2025**	4719	4504	5685	5515	5947	5929	4947	5046	3345	3461	3178	3502	1962	2262	1053	1334	764	1242
**2030**	5064	4799	4647	4465	5536	5439	5687	5800	4596	4863	2952	3231	2614	3126	1436	1857	934	1567
**2035**	6293	5946	4995	4761	4537	4409	5313	5329	5314	5602	4119	4575	2455	2897	1956	2593	1277	2134
**2040**	4739	4282	6216	5903	4890	4706	4375	4327	4994	5157	4803	5286	3504	4149	1865	2417	1815	3027
**2045**	3982	3473	4687	4253	6100	5840	4734	4625	4141	4199	4551	4882	4134	4816	2750	3526	2055	3425
**2050**	3873	3337	3943	3452	4608	4211	5928	5748	4507	4498	3810	3991	3963	4467	3298	4118	2826	4482

Unit: 10,000 persons

Table S2. Average annual percentage change in stroke incidence rates by sex and age group, 1990–2021

Age group (years)	Sex	AAPC (1990-2021)		
		Ischemic stroke (95% CI)	Intracerebral hemorrhage (95% CI)	Subarachnoid hemorrhage (95% CI)
<5	Total	-1.2570 (-1.4614, -1.0521)	-3.7166 (-3.8127, -3.6205)	-1.9695 (-2.2543, -1.6838)
	Male	-1.4189 (-1.5087, -1.3291)	-4.1273 (-4.2643, -3.9901)	-1.9525 (-2.1027, -1.8021)
	Female	-1.1116 (-1.3752, -0.8473)	-3.3776 (-3.4225, -3.3327)	-1.9553 (-2.1042, -1.8062)
5-9	Total	-0.3824 (-0.6350, -0.1293)	-1.8642 (-2.0133, -1.7149)	-1.1757 (-1.3501, -1.0010)
	Male	-0.4215 (-0.5739, -0.2689)	-2.2250 (-2.3004, -2.1496)	-1.2188 (-1.3337, -1.1037)
	Female	-0.3522 (-0.6599, -0.0436)	-1.5201 (-1.7612, -1.2785)	-1.1034 (-1.2771, -0.9294)
10-14	Total	-0.1696 (-0.2596, -0.0796)	-1.3003 (-1.3774, -1.2231)	-1.0371 (-1.2063, -0.8676)
	Male	0.1271 (0.0868, 0.1674)	-1.4155 (-1.4792, -1.3516)	-1.1174 (-1.2506, -0.9840)
	Female	-0.2662 (-0.3805, -0.1517)	-1.1605 (-1.4304, -0.8899)	-0.9452 (-1.1293, -0.7609)
15-19	Total	0.1174 (0.0731, 0.1617)	-1.0890 (-1.1286, -1.0494)	-1.1297 (-1.2980, -0.9611)
	Male	0.3922 (0.3010, 0.4836)	-1.0146 (-1.1094, -0.9197)	-1.1718 (-1.2753, -1.0682)
	Female	-0.2953 (-0.392, -0.1983)	-1.1607 (-1.2042, -1.1171)	-1.0488 (-1.2373, -0.8599)
20-24	Total	0.2066 (0.1331, 0.2802)	-1.1866 (-1.2316, -1.1416)	-1.2900 (-1.4729, -1.1068)
	Male	0.6630 (0.5520, 0.7742)	-0.7552 (-0.9132, -0.5970)	-1.2566 (-1.3272, -1.1859)
	Female	-0.1569 (-0.1959, -0.1178)	-1.7769 (-1.8473, -1.7065)	-1.3186 (-1.4894, -1.1475)
25-29	Total	0.2552 (0.2095, 0.3010)	-1.3067 (-1.4706, -1.1425)	-1.4365 (-1.5513, -1.3215)
	Male	0.9469 (0.7922, 1.1020)	-0.6119 (-0.7508, -0.4728)	-1.3234 (-1.4086, -1.2380)
	Female	-0.2895 (-0.3575, -0.2214)	-2.5576 (-2.6673, -2.4477)	-1.5992 (-1.7565, -1.4416)
30-34	Total	0.6518 (0.6063, 0.6974)	-1.4102 (-1.5173, -1.303)	-1.7093 (-1.9115, -1.5067)
	Male	1.3432 (1.2619, 1.4245)	-0.6425 (-0.7551, -0.5297)	-1.5997 (-1.7218, -1.4774)
	Female	-0.4949 (-0.6561, -0.3334)	-2.8901 (-3.0204, -2.7596)	-1.8413 (-1.9818, -1.7007)
35-39	Total	1.0270 (0.9321, 1.1219)	-1.4422 (-1.4957, -1.3887)	-1.9519 (-2.1246, -1.7789)
	Male	1.6348 (1.5129, 1.7569)	-0.6546 (-0.8166, -0.4924)	-1.8826 (-1.9812, -1.7839)
	Female	0.4235 (0.3449, 0.5022)	-2.9934 (-3.0633, -2.9235)	-2.0335 (-2.1179, -1.9491)
40-44	Total	1.1846 (1.0860, 1.2833)	-1.8433 (-1.9968, -1.6894)	-2.2888 (-2.4434, -2.1340)
	Male	1.6826 (1.5498, 1.8156)	-1.1353 (-1.2157, -1.0548)	-2.1177 (-2.4162, -1.8182)

Age group (years)	Sex	AAPC (1990-2021)		
		Ischemic stroke (95% CI)	Intracerebral hemorrhage (95% CI)	Subarachnoid hemorrhage (95% CI)
	Female	0.6093 (0.5095, 0.7092)	-3.1087 (-3.2101, -3.0071)	-2.4717 (-2.6647, -2.2783)
45-49	Total	1.2326 (1.0975, 1.3678)	-2.1861 (-2.2968, -2.0753)	-2.5544 (-2.6474, -2.4613)
	Male	1.6901 (1.5395, 1.8410)	-1.5776 (-1.7513, -1.4035)	-2.2679 (-2.3901, -2.1456)
	Female	0.6505 (0.5604, 0.7407)	-3.1323 (-3.2330, -3.0314)	-2.8362 (-3.0935, -2.5782)
50-54	Total	0.9582 (0.7809, 1.1358)	-2.1569 (-2.2275, -2.0863)	-2.6742 (-2.7691, -2.5793)
	Male	1.2700 (1.1507, 1.3894)	-1.7495 (-1.8643, -1.6346)	-2.4200 (-2.5444, -2.2954)
	Female	0.5795 (0.534, 0.6251)	-2.7511 (-2.8751, -2.6270)	-2.9543 (-3.0354, -2.8730)
55-59	Total	0.7627 (0.6590, 0.8665)	-1.9332 (-2.0507, -1.8155)	-2.7370 (-2.8513, -2.6225)
	Male	0.9844 (0.8568, 1.1122)	-1.7617 (-1.8553, -1.6680)	-2.5924 (-2.7263, -2.4583)
	Female	0.5416 (0.4776, 0.6056)	-2.1287 (-2.2547, -2.0026)	-2.8889 (-3.0046, -2.7731)
60-64	Total	0.7518 (0.6510, 0.8527)	-1.9587 (-2.0134, -1.9039)	-2.9143 (-3.0132, -2.8153)
	Male	0.8628 (0.7519, 0.9737)	-1.8625 (-1.9155, -1.8095)	-2.8185 (-3.1297, -2.5062)
	Female	0.6511 (0.5929, 0.7093)	-2.0967 (-2.1994, -1.9940)	-2.9684 (-3.0533, -2.8834)
65-69	Total	0.7411 (0.6899, 0.7924)	-2.0041 (-2.1090, -1.8990)	-3.0648 (-3.1844, -2.9452)
	Male	0.7582 (0.6714, 0.8452)	-1.8753 (-2.0009, -1.7496)	-3.0333 (-3.4996, -2.5647)
	Female	0.7184 (0.5829, 0.8541)	-2.1995 (-2.2775, -2.1213)	-3.1019 (-3.1849, -3.0188)
70-74	Total	1.1371 (1.0594, 1.2147)	-1.8497 (-1.9657, -1.7336)	-3.0497 (-3.3697, -2.7287)
	Male	1.2189 (1.1676, 1.2703)	-1.6439 (-1.7855, -1.5021)	-2.8808 (-3.2313, -2.5291)
	Female	0.9536 (0.8743, 1.033)	-2.2400 (-2.3406, -2.1393)	-3.4013 (-3.5445, -3.2579)
75-79	Total	1.5327 (1.4851, 1.5804)	-1.6797 (-1.7843, -1.5751)	-3.0225 (-3.2987, -2.7456)
	Male	1.6536 (1.5537, 1.7535)	-1.4237 (-1.5320, -1.3154)	-2.7138 (-3.0071, -2.4197)
	Female	1.2436 (1.1296, 1.3577)	-2.2148 (-2.3012, -2.1283)	-3.6444 (-3.8334, -3.4551)
80-84	Total	1.2793 (1.1874, 1.3712)	-1.8203 (-1.9112, -1.7292)	-2.9995 (-3.2036, -2.7949)
	Male	1.4452 (1.3769, 1.5136)	-1.5809 (-1.6836, -1.4781)	-2.7169 (-2.9538, -2.4795)
	Female	1.0019 (0.8799, 1.1241)	-2.1328 (-2.2128, -2.0528)	-3.4310 (-3.6288, -3.2329)
85+	Total	0.6664 (0.5156, 0.8175)	-1.9103 (-1.9989, -1.8217)	-2.9652 (-3.2419, -2.6877)
	Male	0.6277 (0.5978, 0.6577)	-1.8915 (-2.1076, -1.6749)	-2.9379 (-3.1370, -2.7383)
	Female	0.6951 (0.5131, 0.8773)	-1.9417 (-2.0270, -1.8563)	-3.0217 (-3.2627, -2.7802)

Table S3. Summary of methodological framework and key assumptions

Model	Brief description	Main data	Output or key assumption
Study objective and perspective	To project age- and sex-specific incident cases of ischemic stroke (IS), intracerebral hemorrhage (ICH), and subarachnoid hemorrhage (SAH) in China for 2022-2050, and to estimate annual costs from the hospital perspective, healthcare system perspective, and societal perspective over a 12-month time horizon.	GBD 2021 stroke burden data; Chinese stroke cost literature; national health statistics	National projections of stroke burden and costs by stroke subtype, sex, and age group (2022-2050)
Incidence projection	To estimate age-, sex-, and subtype-specific AAPCs using Joinpoint regression, and project future incidence rates from the 2021 baseline.	GBD 2021 age-, sex-, and subtype-specific stroke incidence rates (1990-2021)	Projected age-, sex-, and subtype-specific stroke incidence rates for 2022-2050
Annual incident cases	To combine projected incidence rates with age- and sex-specific population projections to calculate annual new stroke cases.	CPDRC/UNFPA medium-variant population projections for China[10]	Annual incident stroke cases by subtype, sex, and age group (2022-2050)
Cost standardization	To standardize source costs to 2021 US dollars using a two-stage computation, adapting the CPI for inflation adjustment and PPP for currency conversion.	National CPI data; OECD PPP exchange rates[14]; original cost data from literature[12,15-16]	Comparable unit costs across different data sources, adjusted to 2021 USD
12-month stroke total cost model	To construct a subtype-specific decision tree with three clinical pathways: survival without readmission, survival with readmission, and in-hospital death, to estimate 12-month post-stroke hospitalization costs.	In-hospital mortality rates; 12-month readmission probability; unit costs of stroke hospitalization and readmission from Chinese clinical and cost literature[5,11,17-19]	Expected 12-month hospitalization cost per hospitalized stroke case, by subtype, sex, and age group
Cost scaling	To expand hospitalization costs to direct medical costs and total societal costs (including indirect costs) using literature-based cost-component ratios from long-term follow-up studies.	long-term follow-up study data on cost components[13]	Base-case direct medical costs multiplier; Base-case societal costs multiplier
Sensitivity analyses	To vary baseline parameters (incidence rate, mortality, admission/readmission/recurrence rate, cost multipliers) in one-way sensitivity	Scenario analyses	Robustness of projected stroke incidence and cost estimates; uncertainty quantification for key outcomes

Model	Brief description	Main data	Output or key assumption
	analyses, and conduct scenario analyses to test the robustness of projections.		

AAPC, average annual percent change; CPDRC, China Population and Development Research Center; GBD, Global Burden of Disease; ICH, intracerebral hemorrhage; IS, ischemic stroke; SAH, subarachnoid hemorrhage; UNFPA, United Nations Population Fund; OECD, Organisation for Economic Co-operation and Development.

Table S4. Empirical cost composition and cost ratios relative to hospitalization cost

Cost component (1-year, IS)[13]	Cost per patient (CNY)	Ratio vs hospitalization cost
Hospitalization cost	18,143.79	1.00
Direct medical cost	26,612.67	1.47
Direct non-medical cost	2,787.56	0.15
Indirect cost	28,167.25	1.55
Total societal cost	57,567.48	3.17

Table S5. Sensitivity analysis of projected incident stroke cases in China, 2022–2050 (Unit: million)

Scenario	Uncertain input varied	Assumption/Range	Stroke type	2022	2025	2030	2035	2040	2045	2050
Base-case	—	Point estimates	IS/ICH/SAH	2.96/1.24/0.15	3.40/1.29/0.15	4.21/1.39/0.14	5.09/1.47/0.14	6.09/1.55/0.13	7.10/1.58/0.12	7.89/1.59/0.11
Incidence trend-low	AAPC	Lower 95% CI	IS/ICH/SAH	2.96/1.24/0.15	3.38/1.29/0.14	4.18/1.37/0.14	5.03/1.45/0.13	5.99/1.52/0.12	6.95/1.54/0.11	7.69/1.54/0.10
Incidence trend-high	AAPC	Upper 95% CI	IS/ICH/SAH	2.97/1.24/0.15	3.41/1.30/0.15	4.24/1.40/0.14	5.16/1.49/0.14	6.19/1.58/0.14	7.25/1.62/0.13	8.10/1.63/0.12
Baseline incidence-low	IR_{2021}	Lower 95% UI	IS/ICH/SAH	2.10/0.91/0.10	2.41/0.95/0.10	2.99/1.02/0.10	3.65/1.09/0.10	4.38/1.15/0.09	5.12/1.18/0.09	5.73/1.19/0.08
Baseline incidence-high	IR_{2021}	Upper 95% UI	IS/ICH/SAH	4.04/1.64/0.20	4.60/1.71/0.20	5.69/1.83/0.19	6.85/1.93/0.19	8.14/2.03/0.18	9.44/2.06/0.16	10.45/2.06/0.15
Combined-low	AAPC + IR_{2021}	AAPC lower 95% CI & IR_{2021} lower 95% UI	IS/ICH/SAH	2.09/0.90/0.10	2.40/0.94/0.10	2.97/1.01/0.10	3.60/1.08/0.09	4.31/1.13/0.09	5.01/1.15/0.08	5.58/1.16/0.07
Combined-high	AAPC + IR_{2021}	AAPC upper 95% CI & IR_{2021} upper 95% UI	IS/ICH/SAH	4.04/1.65/0.20	4.62/1.72/0.20	5.74/1.85/0.20	6.93/1.96/0.19	8.27/2.07/0.18	9.64/2.11/0.17	10.73/2.12/0.16

IS = ischemic stroke; ICH = intracerebral hemorrhage; SAH = subarachnoid hemorrhage.

AAPC indicates the average annual percent change estimated from the GBD 1990–2021 time series; "low/high" scenarios apply the lower/upper bound of the 95% confidence interval of AAPC. IR_{2021} is the baseline incidence rate in 2021; "low/high" scenarios apply the lower/upper bound of the 95% uncertainty interval (UI) for IR_{2021}.

Projected incident cases were calculated by multiplying projected incidence rates by age- and sex-specific population projections; all other model inputs were held constant across incidence uncertainty scenarios.

Table S6. Projected incident stroke cases in China using 2019 (pre-COVID-19) baseline incidence rates, 2022–2050

Year	IS			ICH			SAH		
	Total	Male	Female	Total	Male	Female	Total	Male	Female
2022	2665035	1579352	1082000	1298371	759788	536880	181427	98898	82333
2025	3034637	1787986	1237876	1363630	792326	567204	178921	97647	80793
2030	3720257	2174377	1528959	1471687	847361	617004	173599	95163	77593
2035	4474732	2600051	1851870	1578516	902722	667296	167183	92231	73939
2040	5294694	3069536	2198976	1671586	952814	709683	159001	88557	69351
2045	6118801	3562654	2529950	1717228	984744	724653	147817	83572	63261
2050	6843723	4004132	2830334	1738374	1002698	734485	135797	77770	57549

Table S7. Projected age-specific incident stroke cases by stroke type and sex in China, 2022–2050

Year	2022									2025								
Stroke type	IS			ICH			SAH			IS			ICH			SAH		
Age(years)	total	male	female	total	male	female	total	male	female	total	male	female	total	male	female	total	male	female
<5	3931	1601	2351	1984	859	1133	1410	723	689	3099	1251	1870	1450	617	841	1088	555	534
5-9	5527	2296	3238	2369	1047	1325	1324	674	650	4718	1947	2784	1934	841	1100	1103	558	546
10-14	5779	2570	3212	2809	1435	1374	1266	667	599	5785	2584	3223	2717	1377	1342	1234	646	588
15-19	5710	2705	3000	3147	1825	1322	1337	728	609	6298	2999	3281	3347	1939	1409	1420	770	651
20-24	6156	3090	3062	3856	2512	1359	1575	878	698	6224	3167	3062	3738	2467	1294	1522	849	674
25-29	8300	4296	4011	5847	4092	1770	2304	1281	1023	7565	4037	3555	5083	3670	1465	1995	1125	871
30-34	16152	8596	7529	13721	10131	3637	4343	2419	1925	13156	7244	5838	10503	8045	2622	3294	1866	1433
35-39	22204	12101	10117	21579	16344	5306	5643	3159	2486	26292	14619	11742	23724	18439	5550	6108	3433	2678
40-44	38851	22461	16413	30153	21978	8243	6720	3747	2975	41502	24392	17204	29404	21938	7717	6465	3630	2840
45-49	74924	44401	30593	47191	32582	14733	9319	5119	4206	70173	42274	28077	39870	28126	12053	7785	4326	3473
50-54	156818	92312	64522	75494	49438	26080	13244	7084	6161	145823	87013	59064	63901	42555	21580	11034	5973	5066
55-59	230498	134771	95992	86833	54392	32574	13344	6939	6407	243374	142998	100861	84520	53131	31571	12671	6608	6066
60-64	246251	140881	105115	76328	46930	29254	9781	5148	4631	356021	203336	152264	101684	62391	38999	12653	6646	6010
65-69	435149	243271	191796	124776	76585	48154	13678	7459	6217	390906	218705	172136	103173	63593	39569	10947	5977	4969
70-74	530228	313470	215679	169141	105884	62835	16759	9997	6724	646003	378556	264058	188350	117323	69858	17986	10665	7212
75-79	501485	310408	190113	179446	112820	66257	16931	10601	6296	640801	394655	242676	208214	130803	76202	18852	11815	6927
80-84	362986	202049	162112	171874	93266	79113	13962	7951	6063	419054	231611	187403	180757	97627	83202	14161	8037	6126
85+	313962	113134	199063	222389	86155	136149	14411	6076	8428	368819	130092	237021	241694	91813	149706	15163	6270	8964
Year	2030									2035								
Stroke type	IS			ICH			SAH			IS			ICH			SAH		
Age (years)	total	male	female	total	male	female	total	male	female	total	male	female	total	male	female	total	male	female
<5	2738	1086	1681	1129	466	673	927	469	460	2641	1035	1639	960	386	585	862	435	430
5-9	3043	1247	1808	1157	491	673	684	343	342	2811	1139	1690	992	410	593	607	301	307
10-14	4877	2186	2738	2164	1078	1090	996	514	483	3179	1439	1786	1333	657	680	622	318	305
15-19	6680	3206	3431	3341	1932	1410	1415	761	656	5713	2749	2910	2690	1544	1145	1137	603	535

20-24	7335	3792	3573	4108	2751	1392	1664	923	742	7815	4109	3763	4081	2777	1350	1644	908	737
25-29	7398	4108	3362	4596	3455	1235	1792	1021	771	8742	4988	3899	5021	3881	1276	1945	1107	837
30-34	11216	6495	4615	8074	6535	1835	2494	1444	1058	11192	6742	4322	7264	6144	1521	2210	1294	926
35-39	20377	11923	8632	16247	13420	3432	4076	2348	1739	17704	10850	7146	12473	10897	2389	3049	1792	1272
40-44	54587	32919	21965	33224	25725	8162	7140	4049	3104	42656	26926	16303	22303	18284	5018	4686	2738	1972
45-49	79783	49330	30902	38176	27875	10954	7315	4139	3205	105257	66661	39550	42416	31993	11575	7976	4587	3439
50-54	124431	75758	49220	46618	31846	15197	7839	4320	3530	139732	86735	54022	44758	31340	14095	7329	4108	3240
55-59	233619	139798	95058	70844	45253	26008	10193	5394	4806	197802	120322	79166	52377	33933	18932	7232	3877	3365
60-64	424864	244012	180788	105878	65289	40320	12546	6623	5942	408630	237970	171585	88852	55523	33321	10025	5363	4696
65-69	563715	312065	250680	129587	79484	49747	13021	7040	5964	675011	374704	299297	135151	83602	51276	12861	6978	5869
70-74	632717	373595	255464	158798	100312	57549	14260	8559	5597	941418	553840	379309	203387	128835	72761	17174	10319	6666
75-79	939596	570739	356747	259965	162214	94151	21973	13718	7952	945311	581832	351688	222708	141807	78010	17573	11228	6121
80-84	616045	339347	274208	227479	122940	103986	16776	9551	7161	906860	496610	402456	286665	154634	130362	19902	11335	8398
85+	475356	164094	309582	273631	102021	171241	16263	6603	9701	670209	231486	436459	338883	126785	211424	19081	7778	11332
Year	2040									2045								
Stroke type	IS			ICH			SAH			IS			ICH			SAH		
Age (years)	total	male	female	total	male	female	total	male	female	total	male	female	total	male	female	total	male	female
<5	2637	1026	1649	845	333	523	831	420	414	2457	948	1547	694	268	437	746	378	372
5-9	2833	1142	1712	927	375	567	587	290	300	2958	1190	1789	898	356	558	589	290	302
10-14	2968	1352	1676	1175	571	610	556	280	276	3024	1393	1706	1131	544	593	542	271	272
15-19	3779	1834	1896	1674	960	714	706	372	336	3579	1746	1776	1493	851	641	628	327	303
20-24	6715	3572	3214	3269	2249	1063	1310	717	594	4462	2416	2109	2026	1417	643	808	441	367
25-29	9339	5484	4079	4958	3948	1190	1908	1086	820	8045	4836	3462	3949	3221	900	1510	855	651
30-34	13490	8351	4960	7895	6893	1545	2366	1383	992	14699	9363	5136	7757	7000	1417	2289	1338	960
35-39	18000	11427	7008	11206	10241	1971	2669	1582	1102	22110	14364	8421	12163	11487	1992	2823	1668	1170
40-44	37367	24572	13627	16784	14498	3475	3447	2065	1411	38301	25956	13488	14779	13307	2849	2967	1803	1196
45-49	82482	54587	29420	27993	22251	7110	5165	3080	2144	72469	49877	24648	20713	17268	4918	3750	2307	1506
50-54	182053	114967	68943	49855	35707	15200	7950	4523	3458	140857	92334	51128	32979	24649	9526	5122	3017	2144
55-59	220390	136194	86812	50958	33463	18146	6752	3664	3102	284842	178422	110680	57509	38194	20222	7313	4008	3324

60-64	346889	204558	143917	65813	41618	24336	7071	3828	3280	387316	231058	158903	64116	40993	23397	6559	3590	3015
65-69	651313	365694	285562	113580	71472	42235	10236	5622	4615	555240	314903	240987	84334	53912	30770	7198	3996	3210
70-74	1156015	686141	459556	214986	138281	75066	17071	10396	6478	1143715	690742	445059	183093	120604	61904	13671	8511	5032
75-79	1458546	901413	535785	292595	188396	99888	21554	13965	7281	1840523	1154367	661564	314394	206890	103662	21621	14359	7020
80-84	909650	508725	394313	246158	136148	109096	16088	9417	6574	1420739	805924	604636	329123	185380	142890	20249	12100	8054
85+	983503	339468	640919	436828	163790	271891	23301	9523	13788	1150679	396571	750746	448935	168560	278910	22687	9289	13382

Year	2050								
Stroke type	IS			ICH			SAH		
Age (years)	total	male	female	total	male	female	total	male	female
<5	2074	794	1315	516	195	331	607	308	303
5-9	2880	1157	1745	812	316	513	551	271	283
10-14	3191	1492	1791	1128	539	595	547	273	276
15-19	3700	1823	1806	1452	828	623	610	316	296
20-24	4245	2331	1989	1797	1273	559	713	386	327
25-29	5359	3318	2257	2432	2044	523	924	523	397
30-34	12918	8423	4315	6147	5704	1054	1787	1039	753
35-39	24553	16347	9132	11934	11665	1817	2699	1592	1121
40-44	47437	32722	16365	15725	14576	2863	3086	1879	1242
45-49	74515	52752	24463	17936	15511	4030	3187	2001	1253
50-54	122204	82737	42715	24462	18985	6725	3699	2246	1498
55-59	218534	141548	81992	38526	26399	13094	4702	2655	2070
60-64	501642	302034	203999	72456	46727	26155	7058	3897	3223
65-69	622059	355927	267554	82292	53377	29492	6651	3729	2937
70-74	1000854	614387	381514	137921	92937	45187	9684	6157	3460
75-79	1870576	1201189	652738	272079	184611	85964	17468	11996	5408
80-84	1788976	1038412	742242	354776	205294	149828	20547	12644	7900
85+	1586335	562690	1017058	543649	210692	330900	26027	11005	15022

Values are numbers of incident stroke cases. Totals may not equal the sum of male and female estimates because projections were calculated separately by sex and rounded.
Abbreviations: IS, ischemic stroke; ICH, intracerebral hemorrhage; SAH, subarachnoid hemorrhage.

Table S8. Projected annual hospitalization costs of stroke by type and sex in China, 2022–2050 (Unit: billion)

Year		IS			ICH		SAH		
	Total cost	Male cost	Female cost	Total cost	Male cost	Female cost	Total cost	Male cost	Female cost
2022	24.65	13.75	10.87	17.95	10.41	7.54	3.49	1.91	1.58
95% Lower Bound	24.19	13.50	10.67	17.59	10.20	7.39	3.15	1.73	1.43
95% Upper Bound	25.10	14.01	11.07	18.32	10.62	7.70	3.82	2.09	1.73
2025	28.23	15.72	12.44	18.75	10.82	7.91	3.44	1.89	1.55
95% Lower Bound	27.71	15.43	12.21	18.37	10.60	7.75	3.11	1.71	1.40
95% Upper Bound	28.75	16.01	12.67	19.14	11.04	8.08	3.77	2.07	1.70
2030	34.99	19.42	15.42	20.07	11.49	8.54	3.34	1.84	1.50
95% Lower Bound	34.34	19.06	15.13	19.66	11.26	8.36	3.02	1.66	1.35
95% Upper Bound	35.63	19.77	15.70	20.48	11.73	8.71	3.66	2.02	1.64
2035	42.34	23.36	18.76	21.34	12.08	9.22	3.22	1.78	1.43
95% Lower Bound	41.56	22.93	18.42	20.90	11.83	9.03	2.91	1.61	1.29
95% Upper Bound	43.12	23.79	19.11	21.77	12.32	9.41	3.52	1.95	1.57
2040	50.61	28.02	22.32	22.43	12.63	9.78	3.06	1.71	1.35
95% Lower Bound	49.68	27.50	21.91	21.97	12.37	9.58	2.77	1.54	1.22
95% Upper Bound	51.54	28.54	22.73	22.88	12.88	9.98	3.36	1.87	1.48
2045	58.99	33.06	25.67	22.90	12.97	9.94	2.86	1.62	1.24
95% Lower Bound	57.90	32.45	25.20	22.43	12.70	9.74	2.59	1.47	1.12
95% Upper Bound	60.08	33.67	26.14	23.37	13.23	10.15	3.13	1.78	1.35
2050	65.61	36.74	28.72	22.99	12.92	10.15	2.62	1.49	1.13
95% Lower Bound	64.40	36.07	28.19	22.52	12.66	9.94	2.37	1.35	1.02
95% Upper Bound	66.82	37.42	29.25	23.45	13.19	10.36	2.86	1.63	1.24

Costs are presented in 2021 US dollars. 95% uncertainty intervals (UIs) are based on the 2.5th and 97.5th percentiles from 10,000 Monte Carlo simulations. IS = ischemic stroke; ICH = intracerebral hemorrhage; SAH = subarachnoid hemorrhage.

PubMed：("stroke"[MeSH Terms] OR "ischemic stroke"[Title/Abstract] OR "intracerebral hemorrhage"[Title/Abstract] OR "subarachnoid hemorrhage"[Title/Abstract]) AND ("China"[Title/Abstract]) AND ("cost"[Title/Abstract] OR "hospitalization cost"[Title/Abstract] OR "inpatient cost"[Title/Abstract] OR "cost of illness"[Title/Abstract] OR "readmission"[Title/Abstract] OR "recurrence"[Title/Abstract] OR "mortality"[Title/Abstract] OR "cost of illness")

Web of Science：TS=(stroke OR "ischemic stroke" OR "intracerebral hemorrhage" OR "subarachnoid hemorrhage") AND TS=China AND TS=(cost OR "hospitalization cost" OR "inpatient cost" OR readmission OR recurrence OR mortality OR "cost of illness")

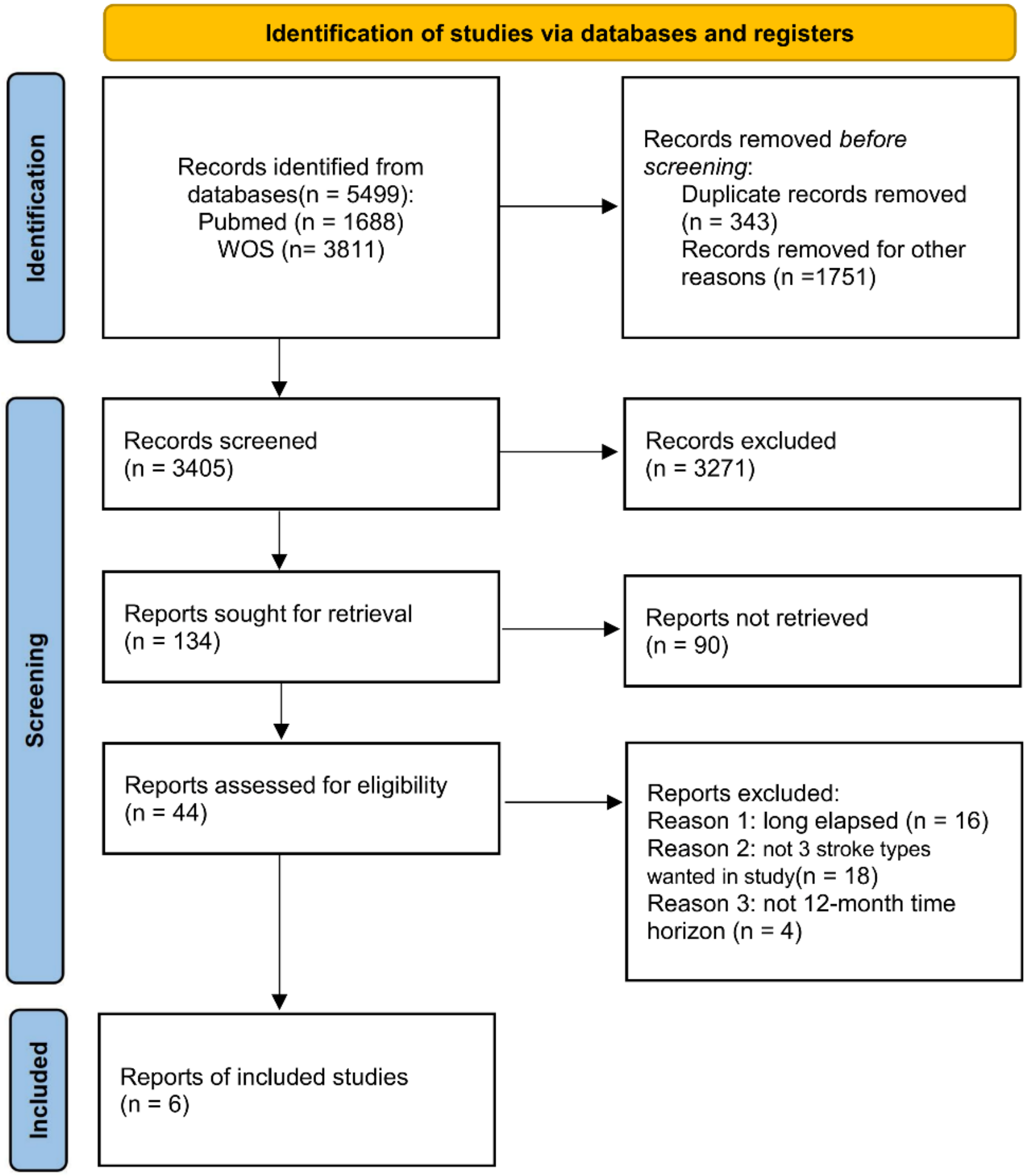

Figure S1. Literature search and screening process for model input parameters

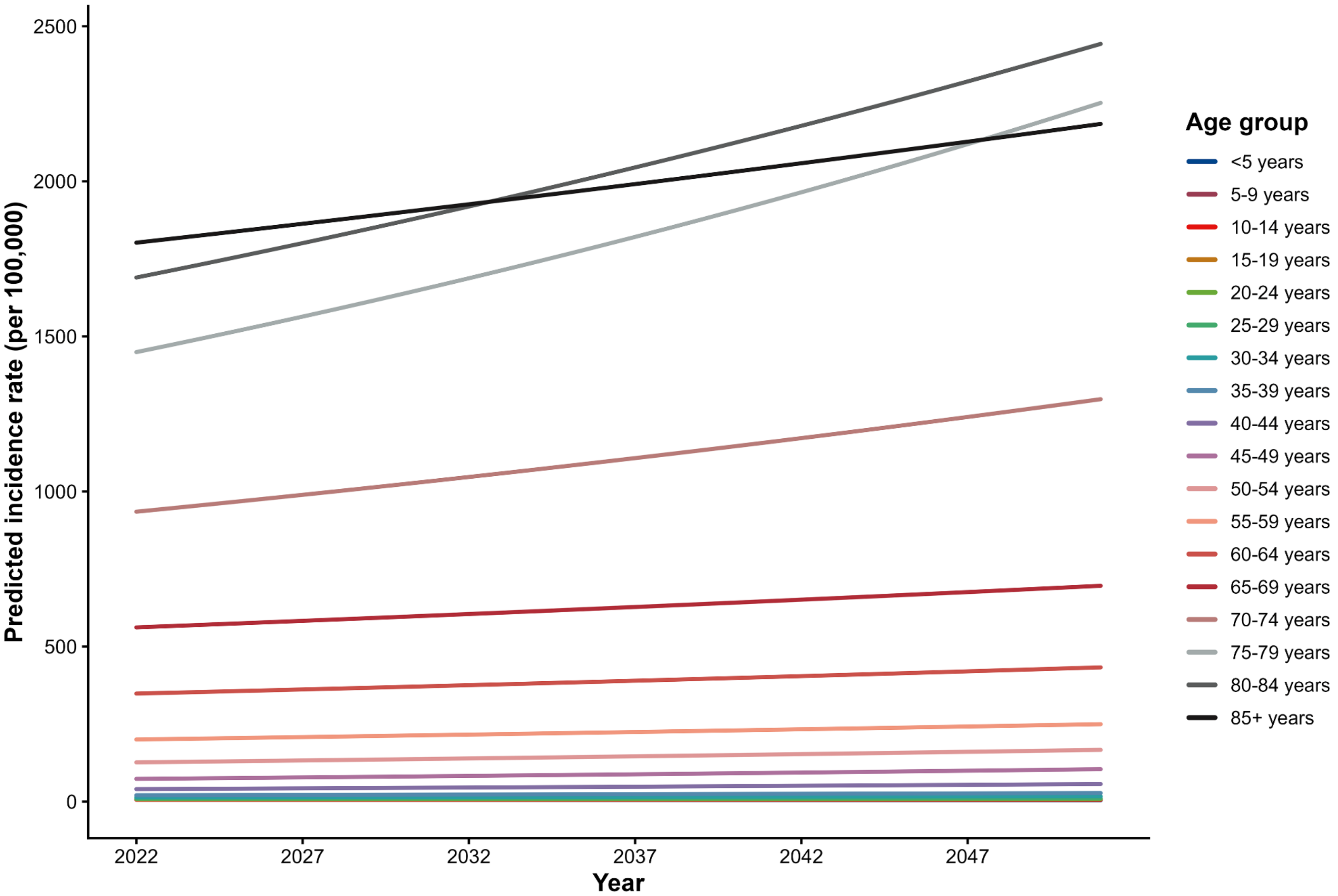

Figure S2. Projected incidence of ischemic stroke by age group, 2022–2050

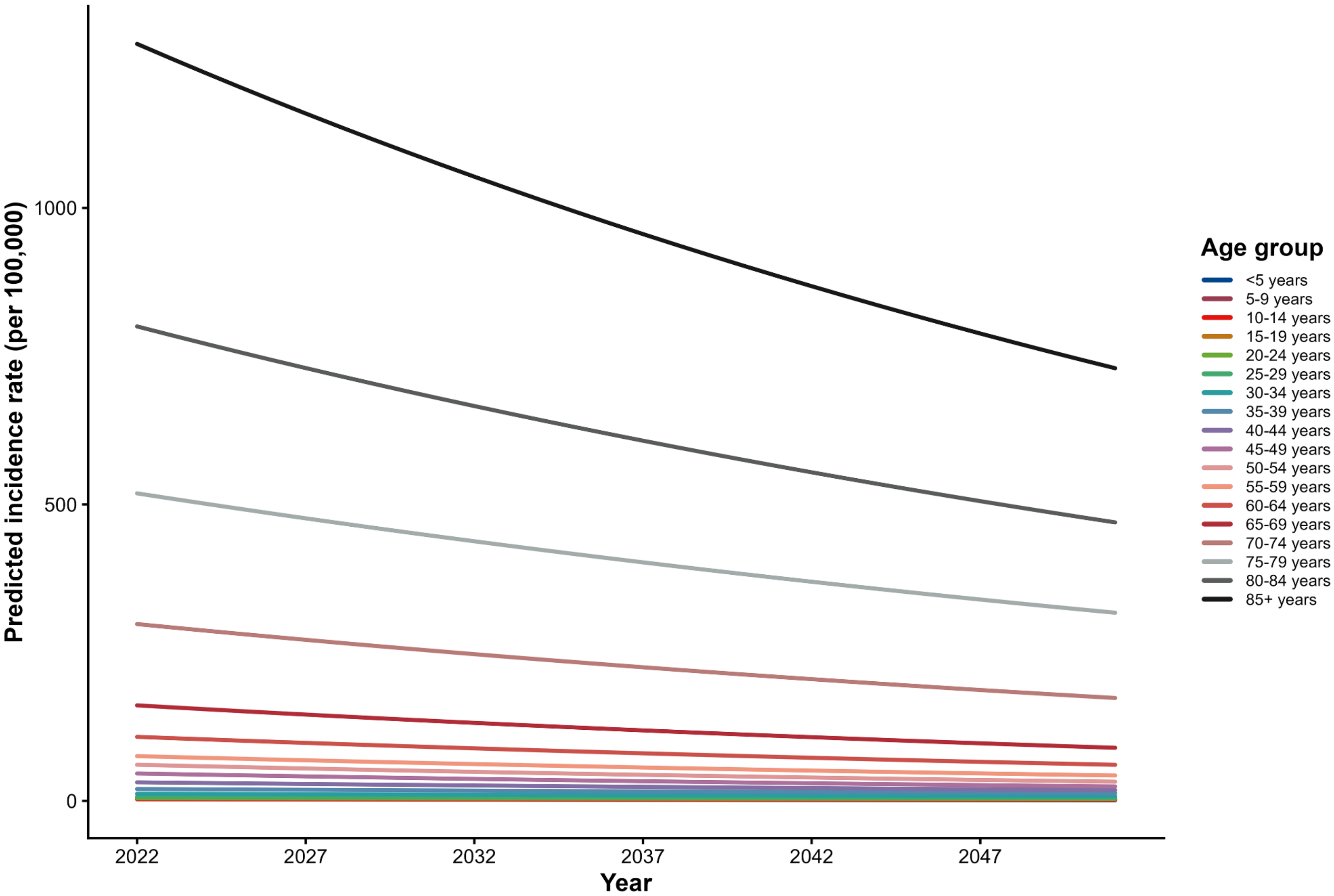

Figure S3. Projected incidence of intracerebral hemorrhage by age group, 2022–2050

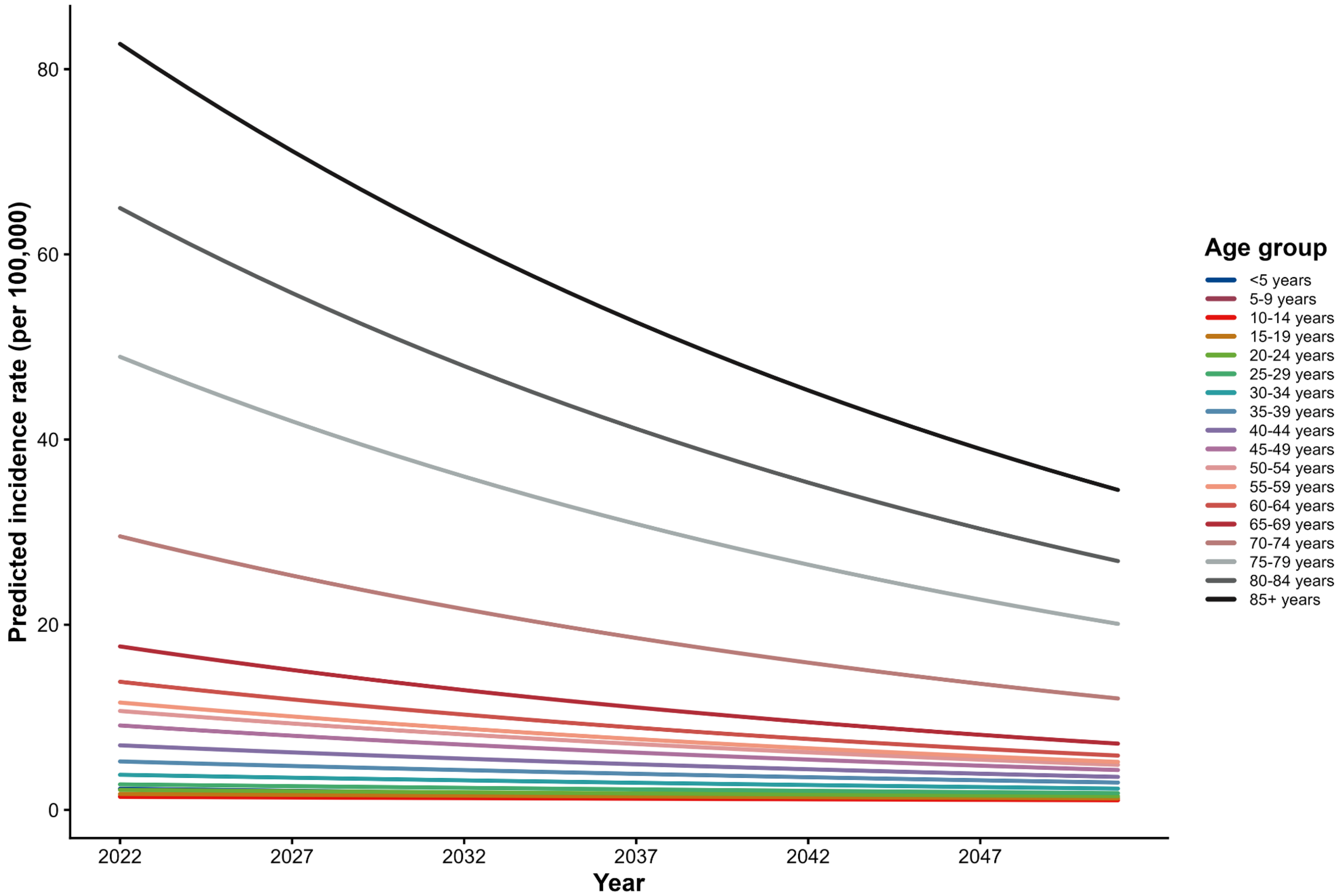

Figure S4. Projected incidence of subarachnoid hemorrhage by age group, 2022–2050

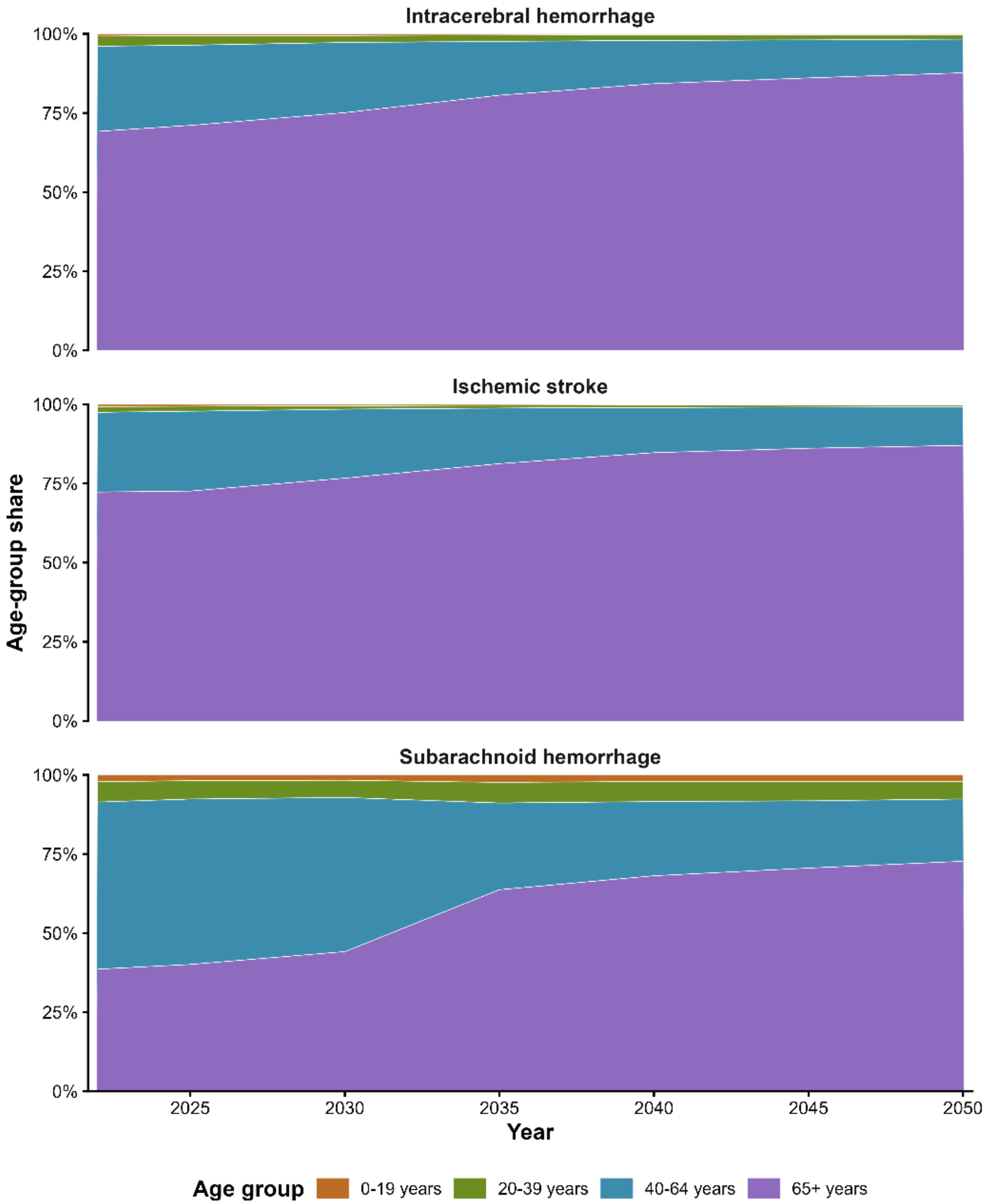

Figure S5. Projected trends in the proportions of stroke incidence by age group, 2022–2050

Consolidated Health Economic Evaluation Reporting Standards (CHEERS) Checklist

	Item	Guidance for Reporting	Reported in section
**TITLE**			
Title	1	Identify the study as an economic evaluation and specify the interventions being compared.	Title
**ABSTRACT**			
Abstract	2	Provide a structured summary that highlights context, key methods, results and alternative analyses.	Abstract section
**INTRODUCTION**			
Background and objectives	3	Give the context for the study, the study question and its practical relevance for decision making in policy or practice.	Introduction, Paragraphs 1–3
**METHODS**			
Health economic analysis plan	4	Indicate whether a health economic analysis plan was developed and where available.	Not applicable; no pre-specified health economic analysis plan documented
Study population	5	Describe characteristics of the study population (such as age range, demographics, socioeconomic, or clinical characteristics).	Section 2.1, Paragraph 1
Setting and location	6	Provide relevant contextual information that may influence findings.	Introduction, Paragraph 2; Section 2.1, Paragraph 1
Comparators	7	Describe the interventions or strategies being compared and why chosen.	Not applicable; this study is a disease-burden and cost-projection study and does not compare interventions or strategies.
Perspective	8	State the perspective(s) adopted by the study and why chosen.	Introduction, Paragraph 3; Section 2.2, Paragraph 2
Time horizon	9	State the time horizon for the study and why appropriate.	Section 2.2, Paragraphs 1-2
Discount rate	10	Report the discount rate(s) and reason chosen.	Not applicable.
Selection of outcomes	11	Describe what outcomes were used as the measure(s) of benefit(s) and harm(s).	Section 2.1-2.2; Section 3.1
Measurement of outcomes	12	Describe how outcomes used to capture benefit(s) and harm(s) were measured.	Section 2.1

Valuation of outcomes	13	Describe the population and methods used to measure and value outcomes.	Section 2.2, Paragraph 2
Measurement and valuation of resources and costs	14	Describe how costs were valued.	Section 2.2, Paragraph 2; Table 1; Supplemental Table S4
Currency, price date, and conversion	15	Report the dates of the estimated resource quantities and unit costs, plus the currency and year of conversion.	Section 2.2, Paragraph 2
Rationale and description of model	16	If modelling is used, describe in detail and why used. Report if the model is publicly available and where it can be accessed.	Section 2.2, Paragraphs 1–2; Figure 1 Table 1; Supplemental Table S3
Analytics and assumptions	17	Describe any methods for analysing or statistically transforming data, any extrapolation methods, and approaches for validating any model used.	Section 2.1–2.4; Supplemental Table S3
Characterizing heterogeneity	18	Describe any methods used for estimating how the results of the study vary for sub-groups.	Section 2.1; Section 3.1; Tables 2–3 Supplemental Table S7–S8
Characterizing distributional effects	19	Describe how impacts are distributed across different individuals or adjustments made to reflect priority populations.	Not formally assessed; descriptive age- and sex-specific distributions of projected cases and costs are reported in Section 3.1 and Tables 2–3.
Characterizing uncertainty	20	Describe methods to characterize any sources of uncertainty in the analysis.	2.3 Sensitivity Analysis; Table 4
Approach to engagement with patients and others affected by the study	21	Describe any approaches to engage patients or service recipients, the general public, communities, or stakeholders (e.g., clinicians or payers) in the design of the study.	Not applicable; no patient engagement in study design
**RESULTS**			
Study parameters	22	Report all analytic inputs (e.g., values, ranges, references) including uncertainty or distributional assumptions.	Sections 2.1-2.3; Table 1; Supplemental Tables S1–S6

Summary of main results	23	Report the mean values for the main categories of costs and outcomes of interest and summarise them in the most appropriate overall measure.	Section 3.1;<br>Tables 2–3;<br>Supplemental Tables S7–S8
Effect of uncertainty	24	Describe how uncertainty about analytic judgments, inputs, or projections<br>affect findings. Report the effect of choice of discount rate and time horizon, if applicable.	Section 2.3; Section 3.2;<br>Table 4;<br>Supplemental Tables S5–S6
Effect of engagement with patients and others affected by the study	25	Report on any difference patient/service recipient, general public, community, or stakeholder involvement made to the approach or findings of the study	Not applicable; no patient involvement
**DISCUSSION**			
Study findings, limitations, generalizability, and current knowledge	26	Report key findings, limitations, ethical or equity considerations not captured, and how these could impact patients, policy, or practice.	Section 4, Discussion; Section 5, Conclusion
**OTHER RELEVANT INFORMATION**			
Source of funding	27	Describe how the study was funded and any role of the funder in the identification, design, conduct, and reporting of the analysis	Sources of Funding
Conflicts of interest	28	Report authors conflicts of interest according to journal or International Committee of Medical Journal Editors requirements.	Disclosures
